# Supplementary material for: Effectiveness and Safety of Ayurvedic Medicines in Type 2 Diabetes Mellitus Management: A Systematic Review and Meta-Analysis
Source: Front Pharmacol. 2022 Jun 8;13:821810. doi: 10.3389/fphar.2022.821810 (PMC9213670; doi:10.3389/fphar.2022.821810)
Supplement: Supplementary file 1 [file DataSheet1.zip › Supplementary Material/Forest and Funnel Plots/Plantago ovata/Plantago ovata.pdf]

Outcomes	Number of studies	Mean Diff. with 95% CI	Heterogeneity (%)
HbA1c	3	-0.87 [-1.41, -0.33]	74.11
FBG	3	-31.50 [-39.83, -23.17]	0.00
Fasting insulin*	2	-3.48 [-6.91, -0.05]	66.62
Fasting C-peptide*	1	-2.50 [-3.22, -1.78]	N/A
Insulin resistance	1	-3.50 [-4.62, -2.38]	N/A
Body weight	1	0.70 [-8.51, 9.91]	N/A
BMI	1	-0.90 [-2.73, 0.93]	N/A
Waist circumference	1	-4.40 [-9.12, 0.32]	N/A
TC	1	-0.30 [-27.13, 26.53]	N/A
HDL-C	1	-6.70 [-12.81, -0.59]	N/A
LDL-C	1	-10.80 [-36.28, 14.68]	N/A
TG	1	-7.40 [-69.90, 55.10]	N/A

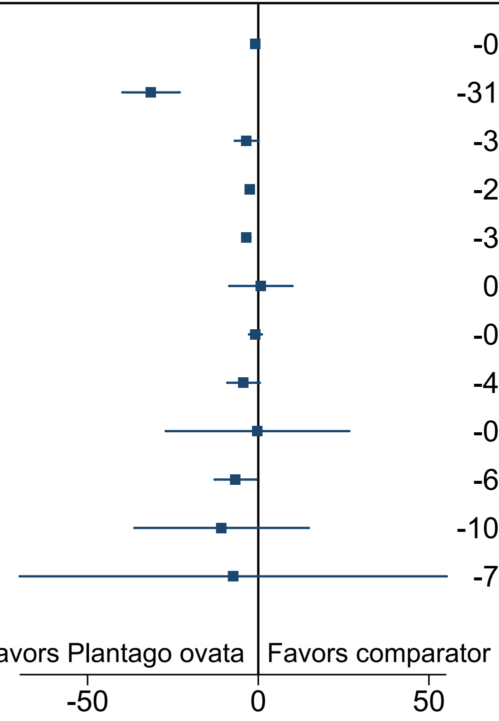

*Favors intervention/comparator as insulin sensitizer
